# Effect of Processing Parameters on the Content of Bioactive Compounds of *Prunus spinosa* L. Fruit Tinctures

**DOI:** 10.3390/foods14183200

**Published:** 2025-09-14

**Authors:** Marta Wilk, Przemysław Seruga, Paulina Nowicka

**Affiliations:** 1 Department of Bioprocess Engineering, Wrocław University of Economics and Business, 118/120 Komandorska Street, 53-345 Wrocław, Poland; przemyslaw.seruga@ue.wroc.pl; 2 Department of Fruit, Vegetable and Plant Nutraceutical Technology, Wrocław University of Environmental and Life Sciences, 37 Chełmońskiego Street, 51-630 Wrocław, Poland; paulina.nowicka@upwr.edu.pl

**Keywords:** *Prunus spinosa* L., tincture, polyphenols, antioxidant capacity, maceration

## Abstract

Blackthorn (*Prunus spinosa* L.) fruits are traditionally used to produce tinctures and are known for their high content of bioactive compounds with antioxidant, anti-inflammatory, and antimicrobial effects. As demand for functional foods increases, it is important to optimize production methods to preserve these health-promoting properties. This study investigated how various parameters influence the polyphenol content and antioxidant capacity of blackthorn tinctures. In the first stage, five ethanol concentrations (40–80% *v*/*v*) were tested, with 80% proving most effective for polyphenol extraction. In the second stage, the influence of fruit pre-treatment (blanched and pricked), sugar addition timing, and maceration duration (28–84 days) was assessed using tinctures made with 80% ethanol. Maximum total polyphenol content was recorded on day 84 in the variant with whole unprocessed fruits, and sugar added at the beginning. Phenolic acids, especially neochlorogenic acid, dominated the extracts. Anthocyanin concentrations reached their maximum during the early phase of maceration and subsequently exhibited a progressive decline over time, likely due to their inherent chemical instability under extraction conditions. Flavan-3-ols and phenolic acids remained more stable, particularly when sugar was added at the beginning. Antioxidant activity strongly correlated with polyphenol content and was highest in samples rich in polymeric procyanidins.

## 1. Introduction

The production of functional food responds to today’s consumer demand. There is a growing search for products rich in bioactive compounds that will positively impact health. There is a revived interest in traditional recipes and natural raw materials. A prime example of this is tinctures made from wild fruits.

Tincture is a traditional Polish spirit whose production began in the 16th century [1]. In the European Union regulations governing the nomenclature and labeling of alcoholic beverages, the term “tincture” is not recognized. In Poland, this term refers to a spirit produced from agricultural ethyl alcohol. The main production stage is the maceration of fruits or plant parts, such as herbs, green walnuts, pine shoots, or flowers (for example, dandelion, elderflower, linden) in ethyl alcohol [2]. Maceration allows the drink to acquire special aromatic properties, and due to the extraction of bioactive compounds, tinctures are also attributed with health-promoting benefits. Until recently, due to a lack of appropriate regulations, alcoholic beverages flavored or made from juices without the maceration process were sold under the name “tincture”. On 19 February 2024, a regulation from the Minister of Agriculture and Rural Development was published in Poland, introducing changes to consumer information rules regarding alcoholic beverages [3]. Considering the traditional production practices commonly applied in the production of “tinctures”, the regulations exclude the possibility of coloring, flavoring, or adding fruit musts, fruit juices, concentrated fruit juices, and wine products. It has been stipulated that production must include a minimum maceration period of 15 days.

The type of raw material used in tincture production and the percentage concentration of alcohol determine the chemical composition of the final product and its potential health effects on consumers. The health-promoting action usually involves supporting treatment or alleviating disease symptoms. For example, drinking 25 mL of blackthorn tincture daily may help combat inflammatory states, regulate digestive issues, or provide relief during colds and flu [2].

Blackthorn (*Prunus spinosa* L.), also known as sloe, is a thorny shrub belonging to the rose family (*Rosaceae*). It is commonly found across Europe, Asia, and northwestern Africa. Its fruiting season occurs in late summer or early autumn, producing small, round stone fruits, blue-black in color, with yellow-green flesh. In traditional medicine, all parts of the blackthorn, including bark, flowers, leaves, fruits, and roots, have been processed and used as natural remedies for treating inflammatory conditions, particularly of the respiratory, urinary, or digestive systems. Currently, the fruits are the most commonly utilized and processed parts, serving as a rich source of vitamins C and A, minerals, as well as phenolic compounds [4,5,6].

Due to the presence of phenolic compounds, including high concentrations of hydroxycinnamic acid, anthocyanins, and flavonol glycosides, blackthorn fruits primarily aid in cancer prevention, intestinal diseases, and the treatment of diabetes, heart disease, cardiovascular disorders, rheumatism, flu, and colds. Additionally, thanks to their polyphenolic content, blackthorn fruits possess confirmed anti-inflammatory, antibacterial, and antioxidant properties, and they also can inhibit activities of enzymes such as α-amylase, α-glucosidase, acetylcholinesterase, and tyrosinase [4,6].

Several studies [5,7,8,9,10,11,12] have investigated the extraction of bioactive compounds from *Prunus spinosa* fruits, typically in the form of fruit powder or homogenate, using complex extraction methods with a variety of solvents, including water, ethanol, acetone, methanol, propylene glycol, dichloromethane, and ethyl acetate, and applying different extraction temperatures. Such processes generally last from several minutes to a maximum of 24 h, and their primary aim is to determine the phytochemical composition of the fruits and/or to assess the biological potential of the extracts, such as antioxidant, anti-inflammatory, or antimicrobial activity. However, these approaches are not applicable to traditional tincture production, which may only employ ethanol as the extraction solvent and, according to legal requirements, must involve a maceration period of at least 15 days.

The type and concentration of bioactive compounds in plant-derived products, including those from blackthorns, depend on the processing method. In tincture production, alcohol concentration as an extracting agent is a decisive factor. The extraction conditions and the recipe used to prepare the tincture also affect the product’s chemical composition.

Reports on quince or cherry tinctures typically apply a single set of processing parameters or focus on differentiating products based on sugar content, often limiting the analysis to total polyphenols [13,14]. A broader qualitative assessment of bioactive compounds was carried out by Barros et al. [15] in grape stem-enriched liqueurs after 90 and 180 days of maceration, yet without introducing variable processing parameters. Regarding *P. spinosa*, Gironés-Vilaplana et al. [16] studied the addition of blackthorn fruits to an aniseed liqueur, applying a 6-month maceration, and performed general phenolic compound analysis every 30 days, with a detailed assessment only at the end of the process, again without varying processing parameters. Similarly, Sokół-Łętowska et al. [17] investigated the composition and antioxidant activity of red fruit liqueurs, including blackthorn, but limited their experimental design to factors such as the absence or presence of sugar (added after 21 days), extraction time, and storage temperature (15 or 30 °C).

In summary, most previous studies have analyzed extraction parameters in isolation, without assessing their combined effects, particularly in the context of traditional tincture production based on maceration. Studies on *P. spinosa* tinctures have not focused on optimizing extraction conditions considering the integrated effect of multiple variables, such as ethanol concentration, raw material pretreatment, timing of sugar addition, and maceration duration, which leaves a research gap. The present study aims to address this gap by systematically evaluating the combined effects of these factors on the polyphenolic composition and antioxidant capacity of traditionally macerated blackthorn (*Prunus spinosa* L.) fruit tinctures.

## 2. Materials and Methods

### 2.1. Reagents and Standards

The following chemical reagents were used for the analyses: 2,2′-azynobis(3-etylobenzotiazolino-6-sulfonian) (ABTS^•+^) from Thermo Fisher GmbH (Kandel, Germany), 2,2-diphenyl-1-picrylhydrazyl (DPPH) and 2,4,6-tris(2-pyridyl)-s-triazine (TPTZ) from Tokyo Chemical Industry Co., Ltd. (Tokyo, Japan), 6-hydroxy-2,5,7,8-tetramethylchroman-2-carboxylic acid (Trolox) from Sigma-Aldrich, Co. (St. Louis, MO, USA), potassium sulfate (K_2_S_2_O_8_), acetic acid (CH_3_COOH), sodium acetate (CH_3_COONa), hydrochloric acid (HCl) from Chem-Pur (Piekary Śląskie, Poland), iron(III) chloride (FeCl_3_) from Sigma-Aldrich Chemie GmbH (Steinheim, Germany), 96% ethanol from Avantor Performance Materials Poland S.A. (Gliwice, Poland). Acetonitrile and methanol used for LC/MS and UPLC analysis were purchased from Merck (Darmstadt, Germany). (−)-Epicatechin, (+)-catechin, cyanidin-3-O-glucoside, cyanidin-3-O-rutinoside, cyanidin-3-O-rhamnoside, quercetin-3-O-rutinoside, kaempferol-3-O-rutinoside, and caffeic acid used for the identification of bioactive compounds in blackthorn tinctures were purchased from Extrasynthese (Lyon Nord, France). Chlorogenic and neochlorogenic acids were provided by TRANS MIT GmbH (Giessen, Germany).

### 2.2. Materials

The fruits (*Prunus spinosa* L.) were obtained from a farm located in the Lublin Province, Poland. After harvesting, the fruits were cleaned, frozen, and stored at −18 °C until tinctures were prepared.

An aqueous ethanol solution made from 95% (*v*/*v*) spirit (Polmos Białystok S.A., Białystok, Poland) and beet sugar of the ‘Polski Cukier’ brand (Krajowa Grupa Spożywcza S.A., Toruń, Poland) were used to prepare the tinctures.

### 2.3. Method of Conducting the Experiment

The study was conducted in two stages.

The first stage aimed to evaluate the effect of ethanol concentration on the total polyphenol content in blackthorn tinctures. The research material consisted of 5 variants of tinctures (V40, V50, V60, V70, V80) differing in the concentration of the aqueous ethanol solution (40, 50, 60, 70, 80 [% (*v*/*v*)]). During the initial stage of the experiment, all samples were subjected to maceration under sugar-free conditions. This approach allowed for the assessment of the extraction process under completely sugar-free conditions prior to the planned supplementation. The fruits of blackthorn and the aqueous ethanol solution were mixed in a 1:1 ratio (*w*/*v*), according to traditional recipes. Each variant of the experiment was performed in two repetitions. The tinctures were kept in the dark at room temperature for 28 days, shaking the mixture every 2 days. After the maceration period, the extract was frozen at −20 °C and stored until analysis. The average values were reported.

The second stage of the research aimed to investigate the influence of processing fruits *Prunus spinosa* L., maceration time, and the sugar addition timing on the content of polyphenolic compounds in the resulting tinctures. The research material consisted of 4 variants of tinctures with an ethanol concentration of 80% *v*/*v* (Table 1). Given the relatively tough skin of blackthorn fruits, preliminary processing was applied to partially break or soften the skin. Accordingly, pricking and blanching were selected to assess whether these treatments could facilitate extraction and enhance its efficiency. Sugar was added, either at the beginning of the process (V2) or on day 56 (V1), only in selected variants (with untreated fruits), in accordance with the experimental design aimed at evaluating the effect of sugar addition timing. A variant entirely free of sugar throughout the maceration period was not included, as the study focused specifically on the scheduling of sugar addition rather than its complete absence. Variants V1, V3, and V4 were designed with sugar addition on day 56 to assess the effect of raw material treatment on polyphenol extraction under standardized conditions. Samples were prepared in 2 repetitions. The fruits, amounting to 500 g, were mixed with the aqueous ethanol solution in a 1:1 ratio (*w*/*v*).

The tinctures were kept in the dark at room temperature for 84 days, shaking the mixture weekly. In each variant, samples were taken on days 28, 56, and 84. The collected samples were stored at −20 °C until analysis. The average values were reported.

### 2.4. Identification and Quantification of Polyphenols

Polyphenols from blackthorn tinctures were analyzed according to the guidelines of Wojdyło et al. [18]. Anthocyanins, flavan-3-ols, flavonols, and phenolic acids were analyzed using ultra-performance liquid chromatography (Acquity UPLC System; Waters Corp., Milford, MA, USA) with a photodiode array detector (PDA), fluorescence detector (FL), and binary solvent manager. Before injection, the tincture samples were centrifuged at 15,000 rpm for 7 min at 4 °C using a Sigma 4K15 centrifuge (Sigma Laborzentrifugen GmbH, Osterode am Harz, Germany). The supernatants were filtered through 0.20 μm hydrophilic PTFE membranes (Millipore Millex Samplicity, Merck, Darmstadt, Germany). The prepared sample was separated in a BEH C18 column (2.1 × 100 mm, 1.7 μm, Waters Corp.; Dublin, Ireland), at a flow rate of 0.45 mL min^−1^ at 30 °C with gradient elution of solvent A (2.0% formic acid) and solvent B (acetonitrile) for a duration of 15 min.

Polymeric procyanidins were analyzed by the phloroglucinolysis method previously described by Wojdyło et al. [18] using a UPLC-FL Acquity system, Waters Corp., Milford, MA, USA (column type, BEH C18 RP, 1.7 μm; column size, 2.1  ×  5 mm; effluent, 2.5% acetic acid/acetonitrile; flow rate, 0.42 mL min^−1^; temperature, 15 °C; wavelength, 360 nm—emission, 278 nm—excitation).

All determinations were performed in triplicate and expressed as mg/L of the sample (anthocyanins, flavan-3-ols, flavonols, and phenolic acids) or mg/100 g dw (polymeric procyanidins). The average values were reported.

### 2.5. Antioxidant Capacity by ABTS^•+^, DPPH, and FRAP Assay

Before analysis, the tincture samples were centrifuged at 15,000 rpm for 7 min at 4 °C using a Sigma 4K15 centrifuge (Sigma Laborzentrifugen GmbH, Osterode am Harz, Germany). The antioxidant capacity was expressed as millimoles of Trolox equivalents (mM TE).

The ABTS radical cation decolorization test described by Re et al. [19] was used to determine the free radical scavenging activity. The stock solution of 2.45 mM ABTS^+^ radicals was prepared by combining 7 mM ABTS and 140 mM potassium sulfate. The solution was left in a dark place for 16 h at 25 °C. A working solution was prepared by diluting the basic solution in 96% ethanol to achieve an absorbance measurement at a wavelength of 734 nm of 0.7 ± 0.02. The antioxidant activity was measured by adding 100 µL of the sample to 1000 µL of the working solution. A blank sample was prepared by mixing 100 µL of 96% ethanol with 1000 µL of the working solution. Measurements were performed spectrophotometrically at 734 nm using a DR 5000™ UV-Vis spectrophotometer (Hach, ON, Canada).

The antiradical DPPH activity of the blackthorn extract was measured following the method described by Klymenko et al. [20]. That is, 0.5 mL of the tested sample was mixed with 3 mL of a 0.1 mM ethanolic DPPH solution to perform the measurement. The samples were incubated in a dark place at approximately 25 °C for 10 min. Measurements were performed spectrophotometrically at 517 nm using a DR 5000™ UV-Vis spectrophotometer (Hach, Ontario, Canada). Measurements were referenced to the blank sample.

The ferric reducing ability of plasma (FRAP) test by Benzie and Strain [21] was used to determine the total antioxidant capacity of the tinctures. The FRAP working solution was prepared from 300 mM sodium acetate buffer (pH 3.6), 10 mM TPTZ in 40 mM HCl, and 20 mM FeCl_3_, mixed in a ratio of 10:1:1 (*v*/*v*/*v*). The tested sample was mixed in a ratio of 1:3 with the working solution, and absorbance was measured at 593 nm using a DR 5000™ UV-Vis spectrophotometer (Hach, ON, Canada).

### 2.6. Statistical Analysis

The results of the conducted research were subjected to statistical analysis in the Statistica program version 13.3 (StatSoft, Kraków, Poland). The normality of the distribution of variables was checked using the Shapiro–Wilk test. Depending on the stage of the study, both one-way and two-way analysis of variance (ANOVA) were performed. Duncan’s post hoc test was applied to identify significant differences between means, with a significance level set at *p* < 0.05. Statistically homogeneous groups are indicated by shared letters in the tables and figures. The Pearson correlation coefficient (r) was used to examine the correlation between variables. The analysis results were presented as the mean of three determinations ± standard deviation.

## 3. Results and Discussion

### 3.1. Phenolic Compounds Profile of Tinctures

In all analyzed tinctures from blackthorn fruits, four groups of polyphenols were identified: anthocyanins < flavan-3-ols < flavonols < phenolic acids. During the initial stage of maceration (measurement on day 28), the tinctures exhibited the same profile of polyphenolic compounds. A significant change occurred in the flavonols profile in samples from days 56 and 84 of maceration, as quercetin-3-O-rutinoside was not detected (Table 2). The dominant compound in blackthorn tinctures throughout the maceration period was neochlorogenic acid.

**Figure 1:**
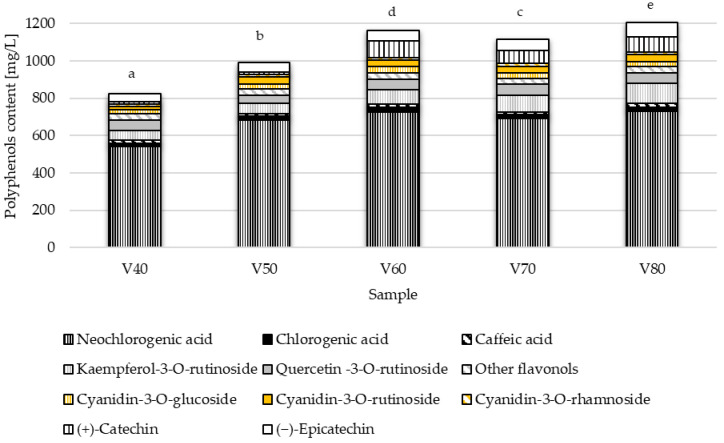
The total polyphenol content (TPC) in tinctures depends on the ethanol concentration. V40–V80 means variant of experiments with 40–80% of ethanol; values marked with the same letter are not significantly different (*p* < 0.05) based on TPC.

The solubility of phenolic compounds depends on the polarity of the solvent, the degree of depolymerization of phenols, the interactions of polyphenols with other components of the plant material, and the formation of insoluble complexes. In studies using fixed extraction conditions, as in the production of tinctures, it is necessary to select the concentration and type of extractant suitable for the specific raw material. The effect of ethanol concentration on polyphenols extraction was tested in five variants of the blackthorn tincture. The results are shown in Figure 1.

Based on the conducted research, it was found that tinctures prepared with ethanol concentrations of 40% and 50% are characterized by the lowest total phenolic compound content (*p* < 0.05). The highest content, amounting to 1204.32 mg/L, was recorded in the tincture with an ethanol concentration of 80% (*v*/*v*) during the first stage of the study. However, if polyphenol groups are considered separately, it should be noted that anthocyanins are an exception, for which it is best to use 60–70% ethanol for extraction. In the group of phenolic acids, similar results were achieved with 60% and 80% ethanol (766.60 mg/L and 770.41 mg/L, respectively). The most significant impact of alcohol concentration on the amount of extracted compounds was observed in the flavan-3-ols group, where the concentration of these compounds in the V40 sample was 57.01 mg/L. In contrast, in the V80 sample, it was 155.11 mg/L. In the flavonols group, the extraction efficiency increased with the rising concentration of the applied alcohol. The obtained results can be explained by the fact that ethanol concentration influences solvent polarity, which in turn affects the solubility of different classes of phenolic compounds. Higher ethanol content can enhance the extraction of less polar compounds, such as hydroxycinnamic acids. The extraction of bioactive compounds from plant materials using organic solvents occurs through mass transfer, including convective diffusion at the particle surface and molecular diffusion within the particles [22]. Effective penetration of the solvent into the plant matrix is essential to intensify this process. Solvent viscosity plays a key role in the rate and extent of compound release. Low viscosity is advantageous because it minimizes molecular resistance and facilitates compound transfer, leading to higher extraction efficiency [23]. The results of the present study confirm that increasing ethanol content in the solvent enhances the concentration of extracted bioactive compounds, likely due to a reduction in solvent viscosity with higher ethanol levels [24].

The effectiveness of high-concentration alcoholic solutions for extracting polyphenols from blackthorn fruit has been confirmed in previous studies. Kotsou et al. [7] obtained the highest total polyphenol yield from *P. spinosa* fruit using a 75% ethanol solution combined with pulsed electric field and ultrasound pre-treatment. Similarly, Magiera et al. [5] used 75% methanol to recover bioactive compounds from *P. spinosa*. However, extraction conditions should be tailored to the specifics of the intended technological process. For instance, Kotsou et al. [7] focused on rapid, technologically assisted extraction of powdered fruit under laboratory conditions at elevated temperature. In contrast, the tincture production process simulated in the present study—featuring prolonged maceration time and sugar addition—required a higher optimal ethanol concentration. Given that the primary aim of the first stage was to identify the ethanol concentration yielding the highest total polyphenolic content, 80% ethanol (V80) was selected for the subsequent experimental stages.

In the second stage of the research, it was checked which method of preparing the raw material would allow for obtaining the most considerable amount of bioactive compounds in the tincture, what effect the maceration time has, and, additionally, whether the timing of sugar addition makes a difference. The quantitative and qualitative characteristics of the extracted polyphenolic compounds in different tincture variants are presented in Table 2. As shown by ANOVA analysis (Table 3), the processing method of blackthorn fruits (variant) and maceration time, as well as their interactions, significantly (*p* < 0.05) influenced the extraction of total polyphenols in the tested tinctures. Additionally, it can be seen that the time of sugar addition is significant (V1 and V2). The greatest extractability of polyphenols was observed in all variants during the first maceration period, over the first 28 days. In the following weeks, the extraction rate was slower. Although the individual increments between days 56 and 84 of maceration differed significantly (*p* < 0.05) in variants V2, V3, and V4, they were only 1.09%, 2.97%, and 6.92%, respectively. Similar observations were made by Narwojsz et al. [14] during the maceration of quince in ethanol, where 81.9% of polyphenols were extracted into the tincture in the first week of the process. The highest total amounts of polyphenols were obtained in the V2 variant, where the fruit was only washed, but sugar was added at the beginning of the process. At the same time, on the 28 day of maceration, the total amount of polyphenols for V2 was the lowest of all variants (up to 12.38%). This can be explained by the “dilution” of the V2 sample due to the 20% (*w*/*v*) sugar addition at the beginning of the process. Similar conclusions were drawn by Sokół-Łętowska et al. [13] when determining the total polyphenols in tincture samples based on cherry juice, for which the difference due to the added sugar averaged 24%.

The dominant group of polyphenolic compounds identified in tinctures at both stages of research was phenolic acids, specifically hydroxycinnamic acids. The extraction of phenolic acids using 40% ethanol was found to be the least effective, averaging 23% (Figure 1). Generally, the longer the maceration lasted, the higher the content of phenolic acids increased (Table 2). The predominant compound among all identified polyphenols extracted from blackthorn fruits was neochlorogenic acid, which accounted for about 60% of the total polyphenolic compounds, which is consistent with other studies [4,7,25]. The presence of chlorogenic and caffeic acids was also noted in the studied tinctures. Research on extracting blackthorn fruits for food was also conducted by Koraqi et al. [26]. Using a solvent system of lactic acid, maltose, and water (66:16:16 *v*/*v*/*v*), they obtained extracts containing phenolic acids (vanillic acid, chlorogenic acid, caffeic acid, and gallic acid) and anthocyanins (cyanidin-3-O-glucoside, cyanidin-3-O-rutinoside, peonidin-3-O-glucoside, and delphinidin-3-O-β-O-glucoside) as the most prevalent bioactive compounds. Stoenescu et al. [27] confirmed the presence of gallic, neochlorogenic, and caffeic acids in dry blackthorn fruit samples extracted with methanol combined with ultrasonic bathing. The differences in the results obtained in our studies can be attributed to the additional identification of chlorogenic acid, but without gallic acid.

On the other hand, Magiera et al. [5] identified neochlorogenic, chlorogenic, and cryptochlorogenic acids in a methanol–water (75:25, *v*/*v*) extract of fresh fruits. Furthermore, protocatechuic, p-hydroxybenzoic, vanillic, and p-coumaric acids were recorded after an additional sequential liquid–liquid extraction using organic solvents. The reasons for the differences in the results of our studies and those of other authors can be sought in the different extractants, the place of fruit collection, the form of the fruit (powdered or fresh), the ratio of fruit to solvent, as well as the conditions and time of extraction.

**Figure 2:**
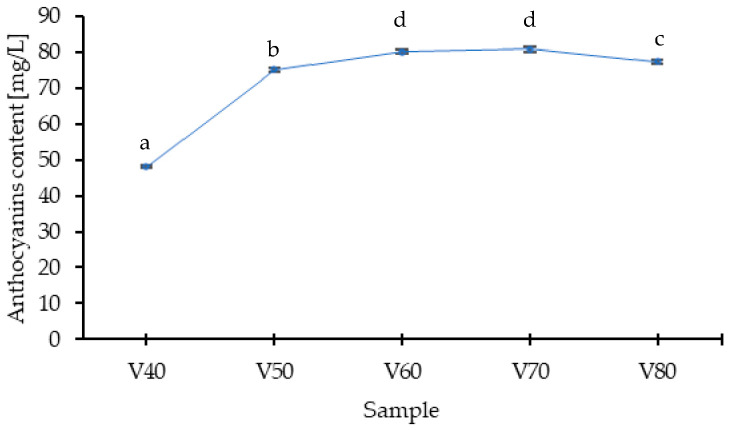
Extraction profile of anthocyanins under varied ethanol concentrations. V40–V80 means variant of experiments with 40–80% of ethanol; values marked with the same letter are not significantly different (*p* < 0.05).

Considering the diversity of the polyphenolic profile of the analyzed tinctures, most compounds identified belonged to the flavonoid class. In the anthocyanin group, three compounds were identified, namely cyanidin-3-O-glucoside and cyanidin-3-O-rutinoside as the dominant ones, and cyanidin-3-O-rhamnoside. The dominant anthocyanins in tinctures are also found in the highest concentration in blackthorn fruits [28]. The content of anthocyanins in the extract increased within the ethanol concentration range from 40% to 70% (Figure 2), with no statistically significant difference in the V60 and V70 samples. Due to their molecular structure (numerous hydroxyl and glycosyl groups), anthocyanins are compounds that dissolve well in polar solvents, so it can be assumed that in solvents containing relatively high amounts of water, extraction will be high. The obtained results indicate that in 40% ethanol, the recovery of anthocyanins is the lowest; this may stem from the specifics of the fruit form used, namely that whole, undamaged blackthorn fruits were subjected to extraction, and in this case, the ethanol concentration was insufficient for effective penetration of the cellular structures. Kotsou et al. [7] studied the key factors influencing the extraction process of bioactive compounds from powdered fruits of *P. spinosa* L. The parameters studied, in addition to temperature and duration of extraction, included the solvent composition (water, ethanol, and their mixtures at 25, 50, and 75% *v*/*v*). These authors proved that lower concentrations of solvent resulted in insufficient extraction, and the optimal conditions for the extraction of anthocyanins were considered to be the use of a 50% ethanol solution at a temperature of 65 °C for 30 min. It should be noted, however, that to achieve optimal extraction, they applied a preliminary ultrasound treatment. This method allows for the rupture of cell membranes, facilitating the extraction of desired compounds.

The anthocyanins are sensitive to many factors, such as pH, storage temperature, copigments, light, sugars and their degradation products, oxygen, enzymes, proteins, and metallic ions [29]. They are therefore unstable compounds, a fact confirmed by the present study. The highest content of these compounds was recorded on the 28 day of the process. Gironés-Vilaplana et al. [16] showed that anthocyanins transfer from fruits to liqueurs within the first 30–60 days of maceration, with maximum anthocyanin levels observed on the 60th day for the liqueur based on *Prunus spinosa* L., after which degradation occurred in the following days. In the presented studies, the concentration of anthocyanins decreased over time during maceration, but starting from the 28 day, which is related to the concentration of the applied solvent (80% *v*/*v*). It has been demonstrated that the anthocyanins’ degradation rate increases with ethanol concentration. The main causes of accelerated anthocyanin degradation in the presence of higher ethanol concentrations include reduced self-association of anthocyanin molecules, which exposes more reactive and unstable forms, and a decrease in the energy of the lowest unoccupied molecular orbital (LUMO) of the flavylium cation. This decrease in LUMO energy facilitates nucleophilic attack by water, leading to hydrolysis of the O-glycosidic bonds at the C-3 position of the flavylium molecule. Additionally, higher ethanol content can shift the equilibrium toward the colorless hemiketal and chalcone forms, further promoting degradation. The combination of these factors—glycosidic bond cleavage, structural rearrangements, and increased susceptibility to hydration—explains the reduced stability of anthocyanins in ethanol-rich solutions [30,31]. The smallest loss of these compounds, at 11.9%, occurred in sample V1-unprocessed fruits, while the largest, at 26.4%, was in V3, where the fruits were blanched.

Among flavonols, the presence of quercetin-3-O-rutinoside and kaempferol-3-O-rutinoside was confirmed, as well as a particular group of unidentified compounds referred to as ‘other flavonols’ (Figure 1, Table 2). The most significant impact of ethanol concentration on flavonol extraction was observed in the case of kaempferol-3-O-rutinoside, which had a concentration of 51.94 mg/L in the tincture produced with 40% ethanol; however, when 80% ethanol was used, it increased to 103.56 mg/L. The increase in quercetin-3-O-rutinoside in these same samples was 11.2%. Interestingly, in the second stage of the study, on days 56 and 84 of the process, the presence of this compound was not recorded, or its quantity was below the detection level of the device. Such changes in the concentration of quercetin-3-O-rutinoside may have been related to polymerization reactions occurring during maceration [15]. The concentration of kaempferol-3-O-rutinoside decreased during maceration by 2–12%, indicating its greater stability. It should also be added that the concentration of quercetin-3-O-rutinoside was, on average, 3.5 times lower than that of kaempferol-3-O-rutinoside (on the 28 day of maceration). Other authors in studies on the extraction of bioactive compounds from *P. spinosa* fruits report the presence of different compounds from this group of flavonoids in the extracts, such as rutin, quercetin 3-O-galactoside, quercetin 3-β-D-glucoside, kaempferol 3-O-β-rutinoside, kaempferol 3-glucoside, avicularin, hyperoside, and isoquercitrin [5,7].

In the analyzed tinctures, monomers of flavan-3-ols in the form of (+)-catechin and (−)-epicatechin were identified. For both compounds, an increase in ethanol concentration improved the effectiveness of extraction, especially at an alcohol concentration ≥ 60%. During the maceration of blackthorn fruits in 80% ethanol, the lowest concentration of (+)-catechin was recorded in sample V1, while the highest was in V2. Still, the maximum increases in the content of this compound were only at the level of 5%. For (−)-epicatechin in samples V2, V3, and V4, a maximum concentration was observed on the 56 day of maceration, with a difference of about 20 mg/L (35%) compared to the 28 day. Interestingly, in tincture V2, to which sugar was added at the beginning of the process, the concentration of flavan-3-ols was the highest (155.65 mg/L), which may indicate the protective effect of sucrose on these compounds. In addition, in sample V1, in which, as in V2, no raw material treatment was applied, but sugar was added at day 56 of maceration, the concentration of flavan-3-ols was the lowest (107.94 mg/L) and at a similar level throughout the maceration period. Shpigelman et al. [32] investigated the protective mechanisms of sugars on epigallocatechin-3-gallate against degradation. They confirmed that sugars (fructose and sucrose) protect the tested compound through multiple mechanisms, including reducing oxygen solubility, chelating transition metal ions, and scavenging reactive oxygen species. Based on these findings, the higher concentration of flavan-3-ols observed in V2 in our study, where sugar was added at the beginning of maceration, may result from the effect of sucrose, which likely reduced oxygen solubility in the solvent and thereby limited oxidative degradation of flavan-3-ols. Additionally, potential chelation of trace metal ions and scavenging of reactive oxygen species by sugar could have contributed to the preservation of these compounds.

**Figure 3:**
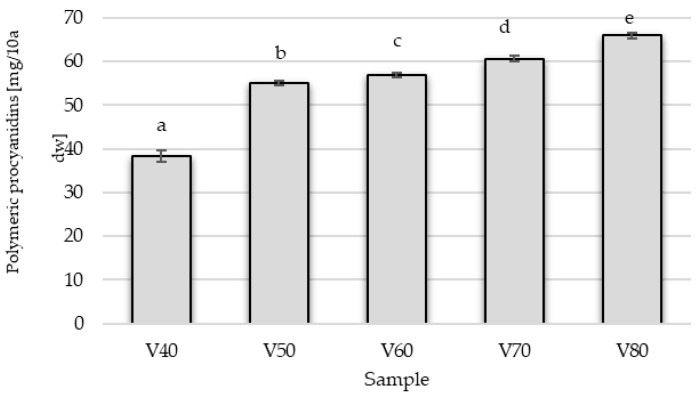
Impact of ethanol concentration on the extraction of polymeric procyanidins in blackthorn tinctures. V40–V80 means variant of experiments with 40–80% of ethanol; values marked with the same letter are not significantly different (*p* < 0.05).

The tinctures were characterized by a low content of polymeric procyanidins (PPCs) with degrees of polymerization (DP) ranging from 1.07 to 1.23, whose concentration was higher when the more concentrated ethanol solution was used for extraction (Figure 3).

Research on fruit juice production has shown that PPCs are bound mainly to insoluble cell wall polysaccharides and do not pass into the juice but remain in the pomace [33]. In tinctures V1 and V2, the fruit has not undergone any treatment, and the extraction of these compounds is lowest (Table 4). The highest concentration of polymeric procyanidins was in the tincture in which the fruit peel was mechanically damaged before maceration (V4). The optimal maceration time for samples in which sugar was added on the 56 day of the process—regardless of the raw material pre-treatment method—was 56 days. In contrast, the sample in which sugar was added at the beginning of the process exhibited the lowest content of polymeric procyanidins. The influence of the tested factors is supported by the statistical analysis (Table 5). It is important to emphasize that the differences between the variants are pronounced and statistically significant, as indicated by a very high F value and a *p*-value below 0.05 (in fact, <0.001). Although Mikulic-Petkovsek et al. [28] reported that blackthorn fruit contains not only (+)-catechins and (−)-epicatechins but also procyanidin dimers and some trimers in the highest amounts, our study demonstrates that primarily flavan-3-ol monomers are extracted into the tincture.

Ethanol, methanol, and water are the most commonly used solvents when extracting polyphenols from a plant matrix for use in food or cosmetic products. Adding water to the extraction mixture increases the polarity of the environment, which enhances the desorption of polyphenols, especially the glycosidic forms, by breaking their hydrogen bonds [34]. Pinacho et al. [12] spoiled blackthorn extracts based on dichloromethane, ethyl acetate, ethanol, and water. They reported the best results regarding total polyphenol content in the ethanol extract. In their study, a solution of ethanol in water was used as a non-toxic substance and approved for tincture production [3].

### 3.2. Antioxidant Capacity of Blackthorn Tinctures

Between the FRAP, DPPH, and ABTS tests, a significantly strong correlation (Pearson correlation (r)) was found in both the first (0.7369 ≤ r ≤ 0.9335) and the second (0.7840 ≤ r ≤ 0.9056) stages of the research.

**Figure 4:**
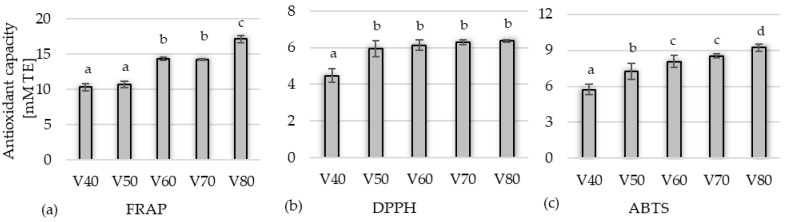
Effect of ethanol concentration (40–80%) on the antioxidant capacity of blackthorn tinctures measured by (**a**) FRAP, (**b**) DPPH, and (**c**) ABTS method. V40–V80 means variant of experiments with 40–80% of ethanol; values marked with the same letter are not significantly different (*p* < 0.05).

The antioxidant activity of the tinctures in the first stage of the study (Figure 4), assessed using the FRAP test, ranged from 10.31 to 17.10 mM TE; for the DPPH test, it ranged from 4.47 to 6.36 mM TE, while for the ABTS test, it ranged from 5.73 to 9.23 mM TE. Generally, the higher the alcohol concentration used to obtain the tincture, the greater activity was observed against Fe^3+^ ions and ABTS•+ cation radicals. Such a correlation was not observed for DPPH, for which the activity did not differ significantly (*p* < 0.05) in the samples from V50 to V80. The antioxidant capacity measured by all methods was strongly correlated with the content of polyphenolic compounds. This study confirms the other reports, e.g., Popović et al. [35] showed that the polyphenols of blackthorn contribute to the overall antioxidant capacity. Pinacho et al. [12] attempted to establish a relationship between antioxidant activity and the nature of active compounds in branches, leaves, and fruits of *P. spinosa* L. The authors concluded that the activity might be mainly attributed to phenolic compounds.

The correlations between the results of the second research stage were considered separately for each variant of the tincture. In the ABTS test, the highest activity was recorded for sample V1 on the 56 day of maceration. This result may have been influenced by the highest anthocyanin content among the other samples, positively correlated with ABTS (r = 0.5648 and 0.7870 for cyanidin-3-O-glucoside and cyanidin-3-O-rutinoside, respectively). Interestingly, despite the highest concentration of polyphenols (Table 2) in sample V2 (sugar was added at the beginning of the process), the antioxidant activity measured by FRAP and ABTS tests was the lowest (Table 6). Based on this, one can infer a strong influence of polymeric procyanidins (r = 0.9786 and 0.8844 for FRAP and ABTS, respectively), whose concentration in this sample was at the lowest level (Table 4). On the other hand, in sample V4, the highest extraction of polymeric procyanidins correlated with the highest activity measured by FRAP and DPPH tests (r = 0.9999 and 0.9729, respectively). In the studied tinctures, an unidentified group of flavonols (‘other flavonols’), caffeic acid, and PPC were significantly positively correlated.

At the same time, quercetin-3-O-rutinoside was significantly negatively correlated with antioxidant activity, regardless of the method of raw material processing. Drăghici-Popa et al. [9] in their research on optimizing extraction conditions of polyphenols from Romanian blackthorn fruits also reported a strongly directed correlation between phenolic acids (caffeic and protocatechuic acids) and antioxidant capacity. In the current study, a strong positive effect of all identified phenolic acids (0.9188 ≤ r ≤ 0.9999), as well as (−)-epicatechin (0.8066 ≤ r ≤ 0.9773), on the formation of antioxidant capacity in tincture sample V2 was observed. In the same sample, contrary to some studies [11,36], correlations between antioxidant determinations and anthocyanins (cyanidin-3-O-glucoside and cyanidin-3-O-rhamnoside), and between ABTS and cyanidin-3-O-rutinoside were inversely correlated (−0.9985 ≤ r ≤ −0.9449). Wojdyło et al. [37], in their studies on 33 different sour cherry cultivars, also suggest that antioxidant activity is not related to the presence of anthocyanins but may be attributed to polymeric procyanidins.

The antioxidant capacity of blackthorn tinctures was significantly affected by both the variant type (raw material pre-treatment and sugar addition timing) and maceration time, as confirmed by two-way ANOVA (*p* < 0.001 for all three assays: FRAP, DPPH, and ABTS) (Table 7). The interaction between these two factors was also statistically significant (*p* < 0.001), indicating that the effect of maceration duration depends on the specific variant.

## 4. Conclusions

This study demonstrates that the ethanol concentration, raw material preparation, sugar addition timing, and maceration duration significantly influence the polyphenolic profile and antioxidant capacity of *Prunus spinosa* L. tinctures. The optimal ethanol concentration for maximizing total polyphenol extraction was 80% (*v*/*v*), using whole, unblanched, and unpricked fruits. Hydroxycinnamic acids were the predominant group of polyphenols. In most experimental variants, the highest total polyphenol content was observed on day 84 of maceration. The highest antioxidant capacity was associated with tinctures containing higher levels of polymeric procyanidins, some flavonols, and phenolic acids. Notably, anthocyanin content declined with prolonged maceration, especially in tinctures made from thermally processed fruits. These findings highlight the importance of control over extraction parameters to enhance the health-promoting qualities of traditional blackthorn-based alcoholic preparations. The study supports the valorization of native fruits and traditional methods in developing functional food products.

Future work should assess the bioavailability of phenolic compounds from black-thorn tinctures in human or animal models. This would provide insight into their real health-promoting effects after ingestion. The study showed that the timing of sugar addition is significant, which indicates a direction for further research. Moreover, future studies could focus not only on the timing of sugar addition but also on a completely sugar-free variant throughout the maceration period, which would help better understand the role of sugar in polyphenol extraction and compound stability.

Tincture preparation is a complex process influenced by numerous interacting factors. This study is limited by the consideration of selected factors influencing the extraction and production process. Future research could explore different *Prunus spinosa* cultivars, seasonal variations, and process scale-up to semi-industrial conditions. Further studies could also address additional variables, such as temperature, extraction method, and pH, to enable more comprehensive process optimization and provide deeper insight into the extraction and transformation of bioactive compounds.

## Figures and Tables

**Table 1:** The tincture variants in the II stage of research.

Variation	Processing Method of Blackthorn Fruits *	Sugar Addition [g]	
		t_0_ **	t_56_ **
V1	-	0	100
V2	-	100	0
V3	Blanching for 15 min at 95 °C	0	100
V4	Pricking	0	100

* excluding cleaning after harvesting; ** t_0_—day of tincture preparation, t_56_—56th day of tincture maceration.

**Table 2:** Polyphenol content in tinctures in the second stage of the research.

Variant of the Blackthorn Tincture *													
Compounds ***		V1-28	V1-56	V1-84	V2-28	V2-56	V2-84	V3-28	V3-56	V3-84	V4-28	V4-56	V4-84
A	C-3-glu	24.06 ± 0.12	24.53 ± 0.12	21.76 ± 0.88	23.33 ± 0.12	18.29 ± 0.14	14.13 ± 0.07	22.62 ± 0.11	18.61 ± 0.09	15.92 ± 0.08	20.26 ± 0.15	20.50 ± 0.10	18.50 ± 0.14
	C-3-rut	23.06 ± 0.14	14.84 ± 0.22	20.92 ± 0.14	21.36 ± 0.19	20.63 ± 0.17	21.19 ± 0.13	24.29 ± 0.22	19.48 ± 0.12	19.18 ± 0.17	26.04 ± 0.22	19.27 ± 0.18	21.81 ± 0.18
	C-3-rha	13.70 ± 0.10	13.76 ± 0.08	11.02 ± 0.06	12.01 ± 0.01	11.45 ± 0.10	9.75 ± 0.07	13.62 ± 0.07	11.55 ± 0.09	9.42 ± 0.05	12.11 ± 0.10	10.08 ± 0.06	8.32 ± 0.07
ΣA		60.82 ± 0.36	63.12 ± 0.42	53.59 ± 0.30	56.71 ± 0.32	50.38 ± 0.41	45.07 ± 0.27	60.53 ± 0.40	49.64 ± 0.30	44.52 ± 0.30	58.41 ± 0.47	49.84 ± 0.33	48.63 ± 0.39
	K-3-rut	190.06 ± 3.27	172.33 ± 0.86	165.40 ± 0.82	186.37 ± 0.93	191.78 ± 0.72	180.99 ± 3.11	188.27 ± 0.94	156.98 ± 2.70	168.30 ± 0.84	181.91 ± 0.68	173.16 ± 0.86	178.42 ± 0.67
F	Q-3-rut	62.51 ± 0.31	0.00	0.00	46.53 ± 0.23	0.00	0.00	62.04 ± 0.31	0.00	0.00	36.30 ± 0.96	0.00	0.00
	Other	27.22 ± 0.16	31.80 ± 0.24	30.51 ± 0.21	20.74 ± 0.15	29.87 ± 0.12	36.83 ± 0.22	24.62 ± 0.18	31.14 ± 0.19	30.45 ± 0.23	30.01 ± 0.12	32.24 ± 0.24	32.17 ± 0.13
ΣF		279.78 ± 3.74	204.13 ± 1.09	195.91 ± 1.04	253.64 ± 1.31	221.65 ± 0.84	217.82 ± 3.33	274.93 ± 1.43	188.11 ± 2.89	198.76 ± 1.06	248.22 ± 1.76	205.40 ± 1.10	210.60 ± 0.80
	NA	533.28 ± 2.65	601.99 ± 2.99	608.01 ± 2.72	431.11 ± 2.15	662.04 ± 3.62	689.10 ± 3.43	467.59 ± 2.33	587.02 ± 2.92	648.62 ± 3.23	517.16 ± 2.83	567.40 ± 2.82	629.01 ± 3.44
P	ChA	17.84 ± 0.10	12.20 ± 0.07	16.28 ± 0.13	16.21 ± 0.10	19.69 ± 0.10	19.35 ± 0.11	17.04 ± 0.10	11.81 ± 0.07	18.62 ± 0.11	16.30 ± 0.08	17.61 ± 0.11	18.50 ± 0.09
	CA	14.37 ± 0.09	20.43 ± 0.18	19.84 ± 0.17	11.17 ± 0.10	20.08 ± 0.09	21.58 ± 0.14	12.72 ± 0.11	18.41 ± 0.12	15.05 ± 0.13	7.53 ± 0.03	17.40 ± 0.16	21.65 ± 0.09
ΣP		565.49 ± 2.84	634.62 ± 3.25	644.13 ± 3.02	458.49 ± 2.34	701.81 ± 3.81	730.03 ± 3.67	497.35 ± 2.54	617.25 ± 3.10	682.29 ± 3.47	541.00 ± 2.94	602.41 ± 3.08	669.16 ± 3.63
F-ols	(+)-Cat	55.86 ± 0.42	58.67 ± 0.32	59.30 ± 0.15	72.491 ± 0.40	74.05 ± 0.44	70.13 ± 0.52	63.49 ± 0.35	58.85 ± 0.44	65.54 ± 0.36	64.45 ± 0.38	53.35 ± 0.29	66.11 ± 0.39
	(−)-Epi	51.84 ± 0.41	48.38 ± 0.24	49.03 ± 0.24	60.75 ± 0.30	82.44 ± 0.40	79.62 ± 0.63	52.98 ± 0.26	72.04 ± 0.57	24.08 ± 0.12	60.90 ± 0.29	80.77 ± 0.40	65.93 ± 0.32
ΣF-ols		107.70 ± 0.83	107.05 ± 0.56	108.33 ± 0.39	133.24 ± 0.70	156.48 ± 0.84	149.75 ± 1.15	116.47 ± 0.61	130.88 ± 1.01	89.61 ± 0.48	125.35 ± 0.68	134.12 ± 0.70	132.04 ± 0.71
TP		1013.79 ± 7.77	1008.92 ± 5.33	1001.96 ± 4.75	902.08 ± 4.67	1130.33 ± 5.89	1142.67 ± 8.43	949.27 ± 4.99	985.88 ± 7.30	1015.18 ± 5.32	972.97 ± 5.85	991.77 ± 5.21	1060.43 ± 5.52
HG **		f	ef	e	a	h	i	b	d	f	c	d	g

* V1–V4 is a type of variant; 28, 56, and 84 are days of maceration. ** HG—Homogeneous groups for total polyphenol content according to Duncan’s multiple range test; Values marked with the same letter are not significantly different (*p* < 0.05). *** A—Anthocyanins; F—Flavonols; P—Phenolic acids, F-ols—Flavan-3-ols; TP—total polyphenols; C-3-glu—Cyanidin-3-O-glucoside; C-3-rut—Cyanidin-3-O-rutinoside; C-3-rha—Cyanidin-3-O-rhamnoside; K-3-rut—Kaempferol-3-O-rutinoside; Q-3-rut—Quercetin-3-O-rutinoside; NA—Neochlorogenic acid; ChA—Chlorogenic acid; CA—Caffeic acid; (+)-Cat—(+)-Catechin; (−)-Epi—(−)-Epicatechin.

**Table 3:** The results of the two-way ANOVA test for total polyphenol in the second stage of the research (*p* < 0.05).

Effect	SS	df	MS	F	*p*
Free parameter	36,998,229.83	1	36,998,229.83	1,036,246.39	0.000
Variant	24,127.59	3	8042.53	225.26	0.000
Maceration time	61,963.36	2	30,981.68	867.73	0.000
Variant × maceration time	77,223.66	6	12,870.61	360.48	0.000

**Table 4:** Polymeric procyanidins content in tinctures in the second stage of the research.

Variant	V1-28	V1-56	V1-84	V2-28	V2-56	V2-84	V3-28	V3-56	V3-84	V4-28	V4-56	V4-84
PPCs	66.70 ^e^	72.58 ^g^	64.15 ^e^	28.08 ^a^	34.22 ^b^	41.38 ^c^	69.85 ^f^	96.21 ^i^	58.76 ^d^	77.17 ^h^	121.22 ^k^	102.06 ^j^
SD	±0.52	±0.67	±0.36	±0.26	±0.27	±0.32	±0.64	±0.75	±0.54	±0.61	±1.11	±0.80

V1–V4 is a type of variant; 28, 56, and 84 are days of maceration. Values marked with the same letter are not significantly different (*p* < 0.05); PPCs—polymeric procyanidins; SD—standard deviation.

**Table 5:** The results of the two-way ANOVA test for polymeric procyanidins in the second stage of the research (*p* < 0.05).

Effect	SS	df	MS	F	*p*
Free parameter	173,324.07	1	173,324.07	68,933.08	0.000
Variant	19,761.28	3	6587.09	2619.77	0.000
Maceration time	2716.56	2	1358.28	540.20	0.000
Variant × maceration time	2851.30	6	475.22	189.00	0.000

**Table 6:** Antioxidant capacity [mM Trolox] of tinctures in the second stage of the research.

Type of Test	Antioxidant Capacity [mM TE] Depending on the Variant of the Blackthorn Tincture											
	V1-28	V1-56	V1-84	V2-28	V2-56	V2-84	V3-28	V3-56	V3-84	V4-28	V4-56	V4-84
FRAP	10.76 ^c^	13.67 ^g^	11.31 ^de^	6.79 ^a^	9.57 ^b^	11.12 ^cd^	9.89 ^b^	14.17 ^h^	12.19 ^f^	11.79 ^ef^	15.32 ^i^	13.74 ^g^
SD	±0.19	±0.22	±0.37	±0.33	±0.35	±0.18	±0.63	±0.43	±0.03	±0.16	±0.40	±0.18
DPPH	4.85 ^b^	6.32 ^f^	5.75 ^de^	3.64 ^a^	5.05 ^bc^	6.35 ^f^	4.88 ^b^	6.16 ^ef^	5.67 ^cde^	5.30 ^bcd^	7.18 ^g^	5.98 ^ef^
SD	±0.37	±0.32	±0.29	±0.43	±0.53	±0.39	±0.36	±0.39	±0.29	±0.25	±0.20	±0.16
ABTS	9.37 ^d^	12.04 ^g^	9.59 ^de^	6.67 ^a^	8.69 ^b^	8.89 ^bc^	9.10 ^c^	9.62 ^d^	9.85 ^de^	10.10 ^e^	11.29 ^f^	9.60 ^d^
SD	±0.49	±0.05	±0.54	±0.16	±0.03	±0.03	±0.19	±0.05	±0.14	±0.14	±0.08	±0.08

V1–V4 is a type of variant; 28, 56, and 84 are days of maceration. Values marked with the same letter in individual rows are not significantly different (*p* < 0.05); SD—standard deviation.

**Table 7:** The results of the two-way ANOVA test for the antioxidant capacity (FRAP, DPPH, ABTS) of blackthorn tinctures in the second stage of the research (*p* < 0.05).

Effect	SS	df	MS	F	*p*
FRAP					
Free parameter	33,599,580.38	1.00	33,599,580.38	63,816.67	0.000
Variant	609,377.35	3.00	203,125.78	385.80	0.000
Maceration time	478,493.89	2.00	239,246.95	454.41	0.000
Variant × maceration time	116,559.25	6.00	19,426.54	36.90	0.000
DPPH					
Free parameter	7,965,724.37	1	79,657,24.37	13,072.48	0.000
Variant	31,722.01	3	10,574.00	17.35	0.000
Maceration time	102,741.22	2	51,370.61	84.30	0.000
Variant × maceration time	42,266.06	6	7044.34	11.56	0.000
ABTS					
Free parameter	22,341,134.98	1	22,341,134.98	81,828.10	0.000
Variant	216,354.79	3	72,118.26	264.15	0.000
Maceration time	91,273.78	2	45,636.89	167.15	0.000
Variant × maceration time	73,775.44	6	12,295.91	45.04	0.000

## Data Availability

All data generated or analyzed during this study are included in this manuscript.
